# Multi-Annual Study of *Eriogaster catax* (Linnaeus, 1758) (Lepidoptera, Lasiocampidae) Oviposition Strategy in Transylvania’s Largest Population: Key Insights for Species Conservation and Local Land Management

**DOI:** 10.3390/insects15100794

**Published:** 2024-10-12

**Authors:** Cristian Sitar, Geanina Magdalena Sitar, Angela Monica Ionică, Vladimír Hula, Lukáš Spitzer, Alina Simona Rusu, László Rakosy

**Affiliations:** 1 Zoological Museum, Babes-Bolyai University, 5–7 Clinicilor, 400006 Cluj-Napoca, Romania; cristian.sitar@ubbcluj.ro; 2 Department of Taxonomy and Ecology, Babes-Bolyai University, 5–7 Clinicilor, 400006 Cluj-Napoca, Romania; 3 Department of Cluj, Emil Racovita Institute of Speleology, Clinicilor 5, 400006 Cluj-Napoca, Romania; 4 Doctoral School “Education, Reflection, Development”, Faculty of Psychology and Educational Sciences, Babes-Bolyai University, 7 Sindicatelor Street, 400029 Cluj-Napoca, Romania; 5 Department of Fundamental Sciences, Faculty of Animal Science and Biotechnologies, University of Agricultural Sciences and Veterinary Medicine Cluj-Napoca, 3–5 Calea Mănăştur, 400372 Cluj-Napoca, Romania; alina.rusu@usamvcluj.ro; 6 Clinical Hospital of Infectious Diseases of Cluj-Napoca, 23 Iuliu Moldovan, 400378 Cluj-Napoca, Romania; ionica.angela@usamvcluj.ro; 7 Department of Forest Ecology, Mendel University in Brno, Zemědělská 1, 613 00 Brno, Czech Republic; hula@mendelu.cz; 8 Museum of the Moravian Wallachia Region, Horní náměstí 2, 755 01 Vsetín, Czech Republic; spitzer.lukas@gmail.com; 9 Biology Centre of the Czech Academy of Sciences, Institute of Entomology, Branišovská 31, 370 05 České Budějovice, Czech Republic; 10 Department of Forestry and Environmental Protection, Stefan cel Mare University, 720229 Suceava, Romania

**Keywords:** *Eriogaster catax*, Lepidoptera, Lasiocampidae, ecology, oviposition strategy, awareness, education

## Abstract

**Simple Summary:**

This study examines the oviposition preferences (egg-laying) of *Eriogaster catax* L., an endangered moth found in warmer parts of Europe, typically in semi-natural areas within agricultural landscapes. Although legally protected in Europe, limited information is available on the species’ specific ecological needs for egg-laying. Our six-year research in Romania reveals that *E. catax* selects blackthorn and hawthorn (*Prunus spinosa* L. and *Crataegus monogyna* Jacq.) shrubs for egg-laying at an average height of 80 cm, with most eggs positioned between 41 and 80 cm above ground. Each cluster typically contains around 186 eggs. The study also provides essential data on the preferred shape and structure of host plants, which serve as a critical food source for larvae and influence the way eggs are laid. This low oviposition height leaves *E. catax* vulnerable to human activities like shrub trimming, burning, and grazing, which can damage or destroy egg sites. These findings support the development of targeted conservation strategies that consider these specific oviposition preferences and highlight the importance of community awareness to protect endangered species and sustain biodiversity.

**Abstract:**

This study provides new insights into the oviposition strategy of *Eriogaster catax* (Linnaeus, 1758) (Lepidoptera, Lasiocampidae), an endangered species of moth found in semi-natural habitats within agricultural landscapes. Protected under various European directives and listed as Data Deficient by the IUCN, *E. catax* inhabits warmer regions of the Western Palearctic. Despite noted geographic variations in its ecological preferences, few studies have statistically significant data on its ecology. Our six-year study, conducted within the largest known population of *E. catax*. in Romania, reveals critical data on its oviposition preferences, including the species’ tendency to utilize *Prunus spinosa* L. and *Crataegus monogyna* Jacq. shrubs at an average height of 80.48 ± 34.3 cm, with most nests placed within the 41–80 cm range and containing an average of 186 ± 22 eggs. The study also addresses the species’ vulnerability to human activities such as bush trimming, agricultural burning, and uncontrolled grazing, particularly due to its low oviposition height. These findings underscore the negative impact of overgrazing and burning practices, particularly when conducted on a large scale, on the conservation of *E. catax.* The detailed ecological requirements identified in this study are essential for developing effective conservation strategies and habitat management practices. Furthermore, the study highlights the importance of local community involvement and public education in raising awareness about biodiversity and the conservation of endangered species.

## 1. Introduction

Over the past decade, public awareness of insect decline has increased significantly [1,2,3]. However, initiatives directed at the general public and educational programs have primarily focused on pollinators, particularly honeybees [4,5,6,7,8], with butterflies also receiving considerable attention in conservation initiatives. This narrow focus on a few well-known species has led to limited conservation actions, overlooking the vast diversity of other insect groups, including many that are key elements in maintaining biodiversity [3].

The decline in insect populations is intrinsically linked to biodiversity loss, which is one of the most pressing ecological issues facing our environment today. In the past thirty years, numerous international initiatives have been established to understand the phenomenon of biodiversity loss and identify solutions to mitigate it [9,10,11]. Despite these efforts, significant results are not yet observed in reversing biodiversity loss trends or in the sustainable exploitation and use of natural resources [9]. One of the primary obstacles in implementing effective solutions is the complexity of the phenomenon, which is only partially understood. Moreover, various stakeholders with specific interests are involved, often directly conflicting with biodiversity conservation goals [12].

A specific example from Romania highlighting this issue is the conservation of Eastern Eggar (*Eriogaster catax* (Linnaeus, 1758)) a species protected by European law. Whereas the species prefers its development overgrown forestless areas, typically pasture grasslands, it is endangered by landowners, who are motivated to clear their land of *Prunus* and *Crataegus* shrubs to receive more subsidies from the Agency for Payments and Intervention in Agriculture (APIA). The preservation of these shrubby biotopes is crucial for the survival of *E. catax* in a wide population. This example underscores the conflict between agricultural policies and biodiversity conservation.

The aim of the present study was to provide new insights into the oviposition strategy of *E. catax* L. This research offers the necessary data concerning the specific preferences with regards to the shape and structure of the host plants, the trophic substrate for the larvae, as well as the way in which the eggs are laid, which may be essential for implementing management and conservation measures.

Considering that this research offers the necessary tools to the local community and stakeholders to find common ground between their own interests and the conservation of this species.

### The Life Cycle, Ecology and Distribution of E. catax

*E. catax* is a species that exemplifies the importance of understanding insect biology, behavior, and ecological requirements in preserving biodiversity. This species, due to its specific ecological requirements and its status as a protected species at the European level, can serve as an umbrella species within its habitat, offering protection to other insect species that use *Prunus spinosa* L. or *Crataegus monogyna* Jacq. as host plants (e.g., *Aporia crataegi* L.; *Iphiclides podalirius* L.; *Odonestis pruni* L.), as well as bird species that nest in these shrublands (e.g., *Acanthis cannabina* L.; *Lanius collurio* L.; *Saxicola rubetra* L., etc.). Additionally, the dense thickets provide small mammals, such as *Muscardinus avellanarius* L., with shelter and food, further enhancing the ecological value of the habitat.

*E. catax* (Lepidoptera, Lasiocampidae) is one of the locally distributed, least studied species [13] and is currently considered an endangered species in several European countries [14], although the risk factors are not well known [15,16]. In Europe, it is also one of the few protected moth species [15] through Annexes II and IV of the Council Directive 92/43/EEC, Annex II of the Bern Convention [17], and, according to the IUCN Red List, it is a Data Deficient (DD) species [18].

*E. catax* is a xero-thermophilic or, by case, a thermo-hydrophilic species, having particular requirements with regards to its habitat [14,16,19,20]. It typically inhabits plain and hilly areas, reaching as far as the submontane region, according to the occurrence of the typical host plants, namely *P. spinosa* and *C. monogyna* [14]. It can be found in natural, semi-natural, and sometimes even anthropic environments [21], including bushy meadows, glades, forest edges, deciduous forests, clearings, or even hedges [17,19,21], but it occurs more frequently in agricultural landscapes with a high diversity of habitats [22], which were formed and maintained by extensive traditional agricultural activities and are characterized by a mosaic aspect and a high biodiversity [23,24,25]. In traditional agricultural landscapes, isolated or grouped bushes are a characteristic element and play an important role as refuge and ecological corridors for several species, including endangered *E. catax* [26,27,28,29,30,31,32]. However, the intensification of agriculture throughout Europe has a strong negative impact on biodiversity [33,34] by loss and destruction of the mosaic of habitats.

**Figure 1:**
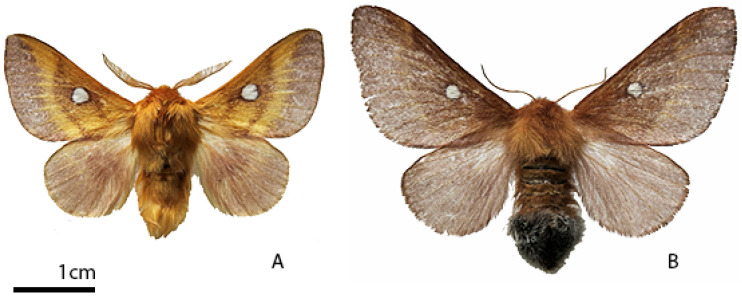
*Eriogaster catax* L. adults: (**A**) male; (**B**) female. Specimens from the Zoological Museum of Babeș-Bolyai University.

**Figure 2:**
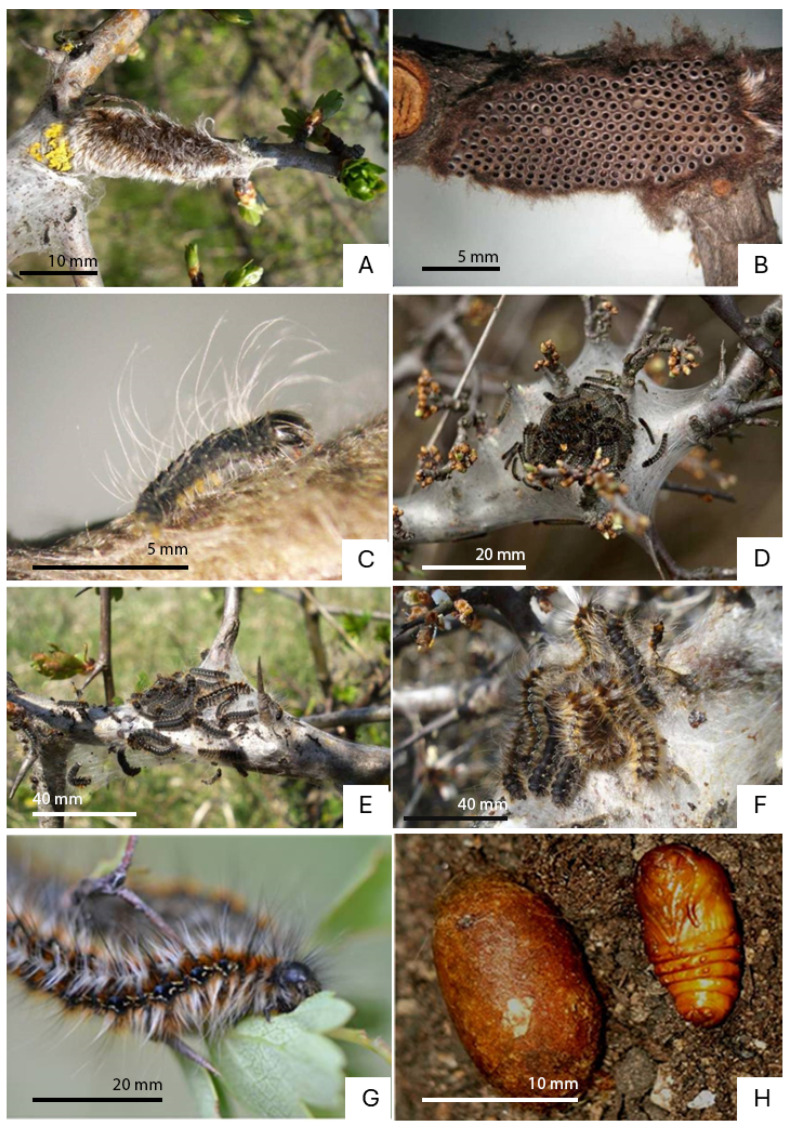
(**A**,**B**) Eggs; (**C**) First instar larvae; (**D**) Second instar larvae; (**E**) Third instar larvae; (**F**) Fourth instar larvae; (**G**) Fifth instar larvae; (**H**) Pupa and Cocoon. Photos taken in situ in the study area—Natura 2000 site The Eastern Hills of Cluj.

The oviposition site selection is crucial for lepidopterans [35], considering that the caterpillars of most species have a reduced mobility; therefore, they require protection from predators and are highly dependent on closely available food sources [36]. The adults of *E. catax* start flying at the end of August [17] (Figure 1), the peak of their flight period having been recorded between the end of September and the end of October, according to weather conditions and geographic area [15,19,20,21,37,38,39,40,41,42]. During this period, the female lays the eggs, preferentially on the branches of *P. spinosa* or *C. monogyna* in most of the areal [43], in a tightly fixed spiral cluster (Figure 2A,B), placed at around 2/3 of the plant’s height [14,15,16,17,19,20,21,38,44,45]. These foodplant species are main across whole Europe with exception of Poland, where the species is reported mainly from *P. spinosa, Pyrus* spp., *Rosa* spp., and then *C. monogyna* [31]. Generally, although the eggs are well camouflaged, they are easy to identify for experienced observers [15].

The embryo overwinters. It lasts for approximately six months, after which the larvae emerge in March or April, according to weather conditions [16,19,38]. One main trait of the larvae (Figure 2C–G) is their social behaviour [38,44]; they are gregarious during the first three developmental stages (Figure 2C–E) [16,19,21,38]. The nest serves as an activity centre and plays an important role in the thermoregulation of the colony [46,47], probably also acting as shelter against predators [46,48]. However, it does not include food resources [48,49,50]; therefore, the larvae leave the platform in order to feed during the warmer period of the day [15,19,20]. The larvae sunbath gregariously mainly during the first three larval instars (Figure 2C–E), mainly for the reason of thermoregulation and also probably as protection against parasitoids [51,52]. The chrysalis stage can be found at ground level (Figure 2H) [15,17,21], under the leaves at the base of the bush, in a silk cocoon [20].

**Figure 3:**
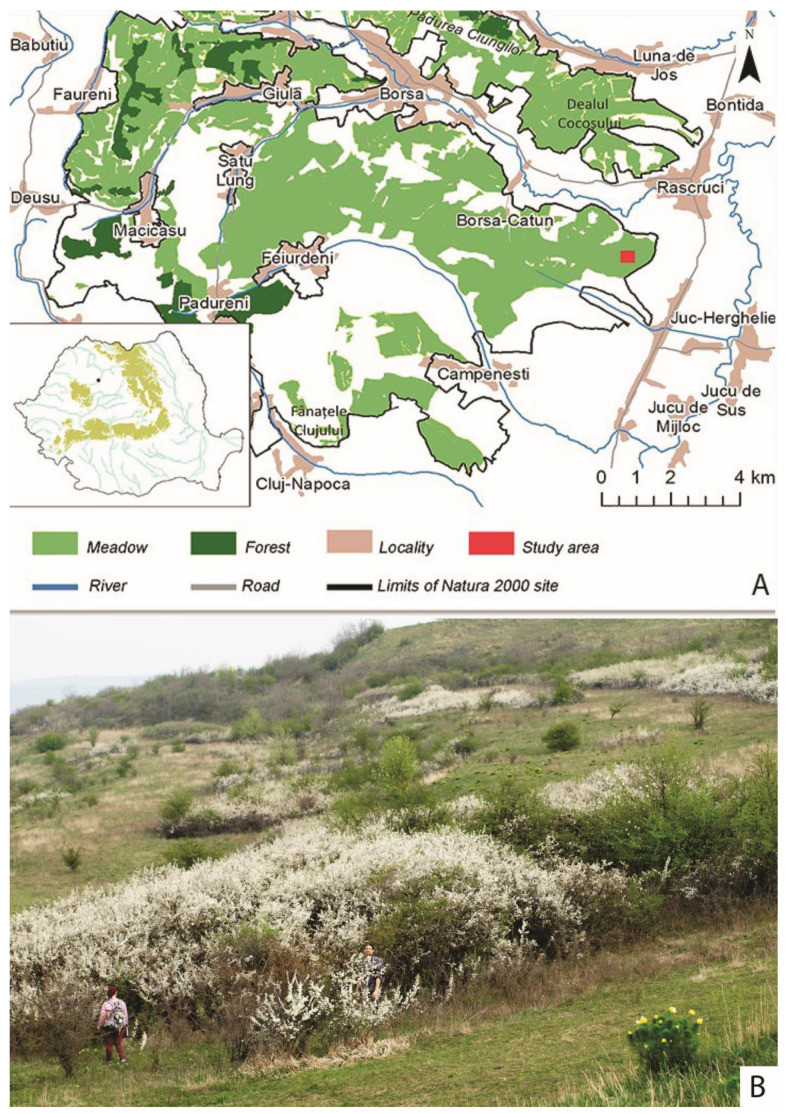
(**A**) Study area in the Eastern Hills of Cluj County. (**B**) Habitat in the study area exhibiting a mosaic structure, characterized by dense clusters of shrubs interspersed with isolated bushes. This spatial arrangement highlights the heterogeneity of vegetation within the landscape.

*E. catax* is present in the warmer regions of the West Palearctic; its distribution ranges from the Iberian Peninsula (Northern Spain) to the Balkans [14,16,19,21] up to Asia Minor and the south of the Ural Mountains [14,16,19,21,48,53,54,55,56]. The species is absent from the Mediterranean islands [14]. In Europe, *E. catax* is currently known from 22 countries: Spain, France, the Netherlands, Belgium, Luxembourg, Switzerland, Italy, Germany, the Czech Republic, Austria, Slovenia, Croatia, Bosnia and Herzegovina, Poland, Slovakia, Hungary, Serbia and Montenegro, Macedonia, Greece, Romania, Bulgaria, Belarus, Ukraine, the Republic of Moldova, and Russia [19,21,56,57,58,59,60]. However, according to [61], this species is not present in Russia or the Ural region. The species forms isolated populations, so that its actual distribution area is in fact discontinuous [21]. With regards to the European distribution, two important centres can be noted, but probably the largest one [16,19,21] is across all Pannonian lowlands (Austria, Slovakia, Czech Republic, Hungary, Croatia, Slovenia, and Serbia). However, during the last century, *E. catax* populations have become extinct in many known locations from Central Europe, including the Bohemia region (part of the Czech Republic) and most of Poland, while their numbers have dramatically decreased in Switzerland and other parts of Europe [53,62,63]. In Romania, *E. catax* occurs in all historical provinces of the country but is rarely reported [64,65] and so far has not been fully mapped at a national level. The largest known population, which was monitored during the present study, is located in Cluj County (Figure 3) in the Eastern Hills of Cluj, ROSCI0295, a Natura 2000 site.

Due to the fact that *E. catax* has become extinct in many regions as a result of habitat destruction for agricultural expansion and the large-scale use of pesticides, the species has been listed in the Annexes of Habitats Directive of the European Union [20]. However, the conservation status of the species remains difficult to determine [17,20,66]. The isolation and numerous population declines from western and central Europe, as well as the existence of populations comprising a small number of individuals, represent impediments in acquiring relevant data regarding the biology and ecology of the species [15,17,21].

## 2. Materials and Methods

### 2.1. Study Area

The present study took place on the surface of the Natura 2000 Site, Eastern Hills of Cluj, ROSCI0295 (Figure 3). The location was chosen based on previous monitoring studies focused on lepidopterans, which revealed a large number of *E. catax* nests. According to [67], the Dealurile Clujului Est Site comprises 5531 hectares of meadows and 405 hectares covered with arboreal vegetation. Furthermore, on most meadows, *P. spinosa* and *C. monogyna* bushes can be found in various proportions [68].

The study perimeters were identified using a Garmin 60 CSx GPS, considering the occurrence of nests, host plants, and land conformation. The areas from which *P. spinosa* and *C. monogyna* were removed by human activity were excluded. Consequently, the study area encompassed 1.896900 square meters (189.69 hectares), located between the villages of Răscruci and Juc-Herghelie, Cluj County, Romania. The population under study is situated at an altitude of 400 m.

### 2.2. Fieldwork and Data Collection

The observations and measurements were carried out between 2011 and 2017, during systematic field trips taking place between March and June in each study year. The year 2017 is not included in the statistic due to only 1 nest observed. Most nests were inventoried during April, when the larvae were in L2 or L3 stage, with the nests already large enough to be easily spotted. All bushes within the perimeter of the site were carefully checked.

The exact position of the nests was recorded using a GPSmap 62s GPS (GARMIN, Olathe, KS, USA). In order to avoid counting the same nest several times and to highlight the smaller nests, they were marked with a coloured plastic ribbon, tied on the tip of the branch. The oviposition height was measured from ground level to the median part of the nest using a 5 m tape measure. The height of the host plant was also recorded.

For each nest, the species of host plant was recorded. According to shape and size, the bushes were divided into two categories: isolated bushes (having a surface of up to 3–4 m^2^) and grouped bushes (having a surface of more than 4 m^2^).

The area of each shrub within the study perimeter was measured. Using ArcGIS Pro, we determined the total land area covered by shrubs within the study perimeter, as well as the ratio between the area covered by *P. spinosa* and *C. monogyna*.

The cardinal orientation of each location was determined using cardinal degrees, as follows: N: 0–22.5 and 337.5–360; NE: 22.5–67.5; E: 67.5–112.5; SE: 112.5–157.5; S: 157.5–202.5; SW: 202.5–247.5; W: 247.5–292.5; NW: 292.5–337.5. The slope exposition was calculated for each location.

In order to determine the number of eggs laid by the female *E. catax*, the nests were collected after being abandoned by the larvae. As the nest is built around the eggs, by removing the silk span by the caterpillars, the eggs exuviae were isolated. The eggs were cleaned from protective hairs using fine tweezers and a brush and counted under a dissection microscope (OPTIKA SZM-2, OPTIKA: Ponteranica, Italy). In order to avoid errors, the counted eggs were marked. The total number of eggs, number of hatched eggs, and unhatched ones were recorded. From the years 2011, 2012, and 2013, a total of 50 nests/year were examined. For the next years, all nests were taken into consideration, namely 33 for 2014, 12 for 2015, and 22 for 2016.

To determine the impact of fire on the nests of *E. catax*, we conducted observations and measurements on the land area where burning was used as a method for removing shrubs. For 150 randomly selected points, we measured the height at which the flames acted on the shrubs. We selected the maximum burning height of the shrub/shrubs or groups of shrubs, considering that all other surrounding shrubs had been completely destroyed by the fire.

### 2.3. Statistical Analyses

The statistical analysis of data was performed using EpiInfo 7^TM^ software (CDC, Atlanta, GA, USA) and the EpiTools website [69]. For the oviposition height, 20 cm intervals were considered, starting from the ground level to the highest point where a nest was recorded. Thus 14 intervals were obtained (0–20 cm, 21–40 cm, … 261–280 cm). For each interval, the frequency and 95% confidence intervals (CI) were established, and the differences were assessed by means of chi square testing and considered significant at a *p* value lower than 0.05. For the total height of the host plant, 50 cm intervals were used (total 7 intervals), as described above. The possible correlation between the host plant height and the oviposition height was assessed using Spearman’s rank correlation test (Spearman’s rho). For the species and grouping of host plants and for the cardinal orientation, the frequency and 95% confidence intervals (95% CI) were established. Differences were assessed by means of a one-sample chi-square test. For the host plant use, we calculated the chi square test based on an expected ratio of 58:42, considering 58% in favour of *P. spinosa* according to host availability. To determine the preference for the shape of the bushes and to determine the cardinal orientation, an equal proportion was used for expected values.

## 3. Results

During the seven years of study, a total of 592 nests were inventoried: 212 in 2011, 224 in 2012, 88 in 2013, 33 in 2014, 12 in 2015, 23 in 2016, and 1 in 2017. The data were analysed both per year and per the entire study period.

### 3.1. Abundance of P. spinosa and C. monogyna Shrubs within the Study Area and the Host Plant Use Differences

**Figure 4:**
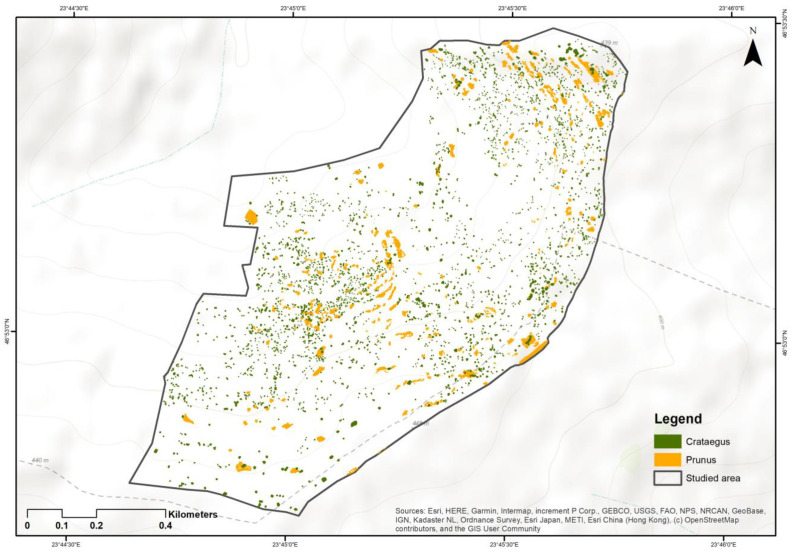
Abundance of *Prunus spinosa* L. and *Crataegus monogyna* Jacq. shrubs within the study area [70].

The area covered by *P. spinosa* is approximately 29,160 square meters, while the area covered by *C. monogyna* is 19,676 square meters (Figure 4). The ratio between the two shrub species is 1.4:1.

Isolated nests (1 or 2) were also seen on *Pyrus ssp*., *Prunus tenella* Batsch, *Rosa canina* L., and *Berberis vulgaris* L.

Overall, the frequency of *P. spinosa* used as the host plant was 54.15% (n = 324), while that of *C. monogyna* was 45.85% (n = 268). The difference was statistically significant (*p* = 0.007; χ^2^ = 7.03; df = 1). The annual values are presented in Table 1.

### 3.2. Shape and Structure of the Host Plant

Overall, 69.88% (n = 414) of nests were found on the grouped bushes (Table 2), indicating a significant preference (*p* < 0.0001; χ^2^ = 188.48; df = 1).

### 3.3. Cardinal Orientation

**Figure 5:**
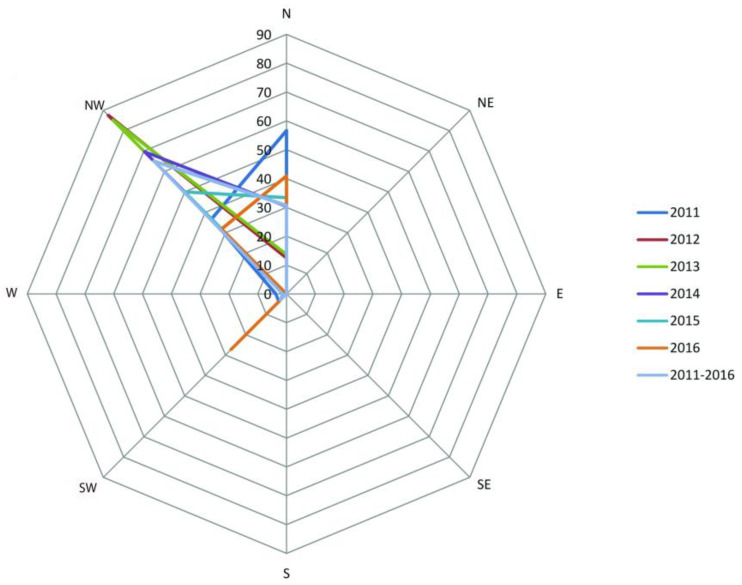
Preference for the cardinal orientation.

A significant majority of nests (65.14%; n = 386) were positioned on the north-western exposition (Figure 5) (*p* < 0.0001; χ^2^ = 884.45; df = 3), followed by the northern one (Table 3).

### 3.4. The Host Plant Height

**Figure 6:**
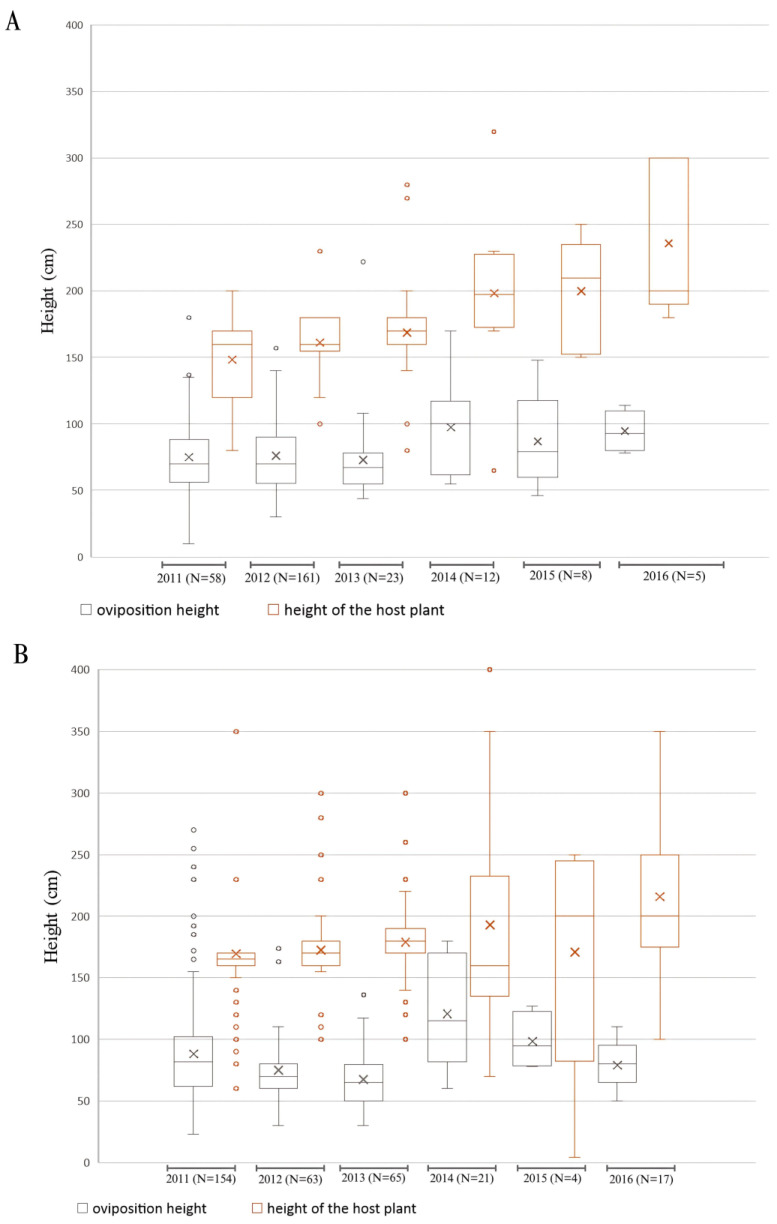
(**A**) Oviposition on *Crataegus monogyna* Jacq. (**B**) Oviposition on *Prunus spinosa* L. Box plots showing the distribution of the oviposition heights (grey) and the host plant heights (orange) from 2011 to 2016. For each year, the oviposition height and host plant height are displayed side by side. The boxes represent the interquartile range (IQR), with the median indicated by the horizontal line and the mean by an “X”. Whiskers extend to the minimum and maximum values within 1.5 times the IQR, and outliers are shown as circles. Across all years, moths predominantly laid eggs at lower heights compared to the overall height of the host plants, with consistent trends in oviposition height despite increasing host plant variability over time.

The host plant height shows a clear difference between the two species, with *C. monogyna* exhibiting more consistent heights across years compared to *P. spinosa*, which shows more variability. For *C. monogyna*, plant heights tend to range between 150 cm and 200 cm, with limited variation over time and a few outliers extending beyond 250 cm (Figure 6A). On the other hand, *P. spinosa* demonstrates wider fluctuations in height, especially in 2015 and 2016, where plant heights range up to 400 cm, with significant outliers (Figure 6B). The greater variability in *P. spinosa’s* height could reflect its growth patterns or environmental influences, which may contribute to the broader range of oviposition sites chosen by the moth.

Analyzing the distribution of the heights of the bushes in 50 cm height intervals, it turned out that most of the bushes are located in the height range of 151–200 cm (see Supplementary Files, Table S1) with statistically significant differences (*p* < 0.0001; χ^2^ = 1465.25; df = 6). Overall, the average height of the host plant was 170.55 ± 48.66 cm (see Supplementary Files, Table S2). The average height of *P. spinosa* was 176.49 ± 54.58 cm and that of *C. monogyna* was 163.33 ± 39.22 cm (see Supplementary Files, Table S2).

### 3.5. Oviposition Height

Over the years, the oviposition height of the moth consistently remains lower than the overall height of the host plant, regardless of whether *C. monogyna* or *P. spinosa* is used as the host (Figure 6). For *C. monogyna*, oviposition heights are more stable across years, with fewer extreme outliers (Figure 6A). When *P. spinosa* is used as the host plant, there is more spread in oviposition height (Figure 6B). In both cases, however, the median oviposition height remains significantly lower than the height of the host plant, suggesting a preference for laying eggs lower on the plant, possibly for reasons related to protection or accessibility for larvae.

Analysing the distribution of oviposition heights on 20 cm height intervals, the most frequent interval was 61–80 cm, followed by 41–60 cm (see Supplementary Files, Table S3). The differences between intervals were statistically significant (*p* < 0.0001; χ^2^ = 1181.66; df = 13).

According to host plant, on *P. spinosa*, the most frequent intervals were 61–80 cm and 81–100 cm (see Supplementary Files, Table S4), with statistically significant differences (*p* < 0.0001; χ^2^ = 694.79; df = 13). On *C. monogyna*, the predilect oviposition intervals were 41–60 cm and 61–80 cm (see Supplementary Files, Table S5), with statistically significant differences (*p* < 0.0001; χ^2^ = 576.90; df = 13). Overall, the average oviposition height was of 80.48 ± 34.3 cm (see Supplementary Files, Table S6). According to the host plant, the average oviposition height was 85.81 ± 39.15 cm on *P. spinosa* and 77.26 ± 28.83 cm for *C. monogyna* (see Supplementary Files, Table S6).

The analysis of possible correlations between the oviposition height and the total height of the host plants revealed strong correlations, but with no statistical significance (R = 0.637748, *p* = 0.173).

### 3.6. Egg Counts

The average number of laid eggs was 186 ± 22 eggs/nest. The maximum number of eggs per nest was 265, while the minimum was 132. Overall, 95% of eggs hatched. The maximum number of unhatched eggs in a single nest was 45, while in three nests, all of the eggs hatched, totalling 215, 218, and 224, respectively.

### 3.7. Anthropogenic Impact on the Dynamics of E. catax Nests from 2011 to 2015

**Figure 7:**
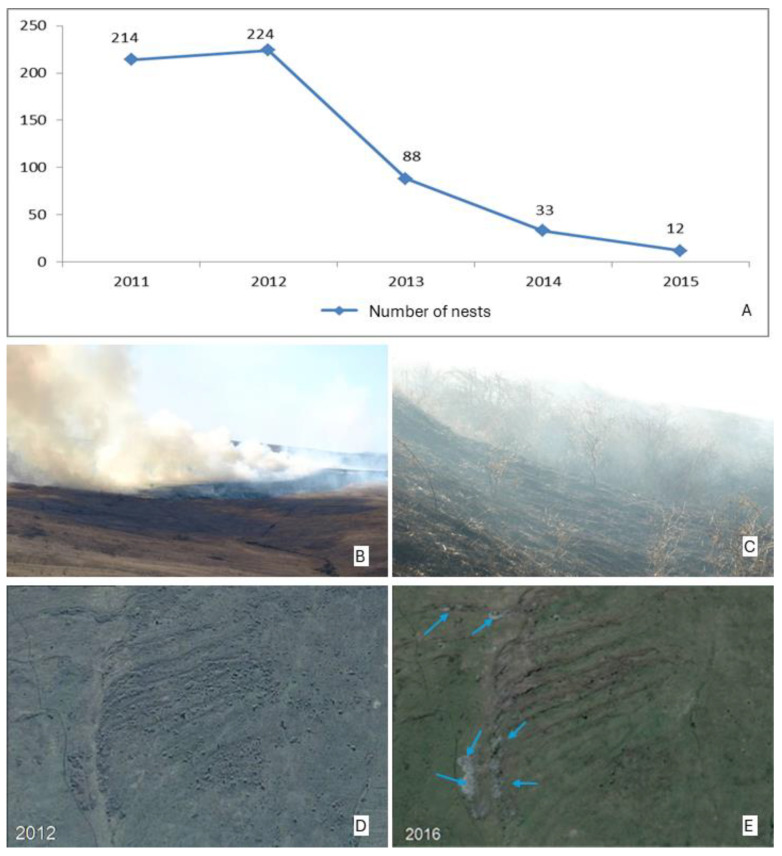
(**A**) Dynamics of *Eriogaster catax* L. nest numbers from 2011 to 2015. (**B**) The direct impact of the fire. (**C**) The direct impact of the fire. (**D**) Coverage of the study area by shrubs exceeding 20% in 2012. (**E**) By 2016, only a few larger shrubs remained, indicated by blue arrows.

Starting in 2013, we recorded a drastic decline in the number of nests and, consequently, in the population of *E. catax* in the study area (Figure 7A). This decline is closely related to the pasture management practices carried out by local farmers.

In 2013, systematic clearing of the pasture began with the removal of shrubs. This clearing was executed through two methods: the burning of the pasture (Figure 7B,C) and mechanized shrub cutting (Figure 7D,E). On the burned area of the study site, the average height of the fire reached 121.41 cm (stdev ± 39.57; n = 150), while the average oviposition height for *E. catax* is 80.48 cm (stdev ± 34.30; n = 592). The average height of shrubs where eggs were laid was 170.55 cm (stdev ± 48.66; n = 224). No nests were found in the area affected by the fire.

Subsequently, mechanical equipment was used to remove the remaining shrubs. By the spring of 2016, approximately 80% of the shrubs had been removed. This pasture management had a direct impact on the *E. catax* population, leading to a negative outcome that significantly reduced the population size.

## 4. Discussion

Previous studies have provided some data on the biology and ecology of *E. catax* [14,15,16,19,20,21,46,48], emphasizing the low density and vulnerability of *E. catax* populations. In Hungary, [71] revealed the existence of a large number of nests near Vát. However, for that population, data such as oviposition height, species of the host plant, shape and size of the host plant, etc., were not recorded. The first studies to statistically approach the oviposition preferences of *E. catax* were recently published from Poland [31] and Romania [72], highlighting that the oviposition preferences are influenced by regional conditions, such as the availability of potential host plants and local climatic factors. However, current knowledge remains limited, making it difficult to generalize these findings across the entire distribution range of the species.

*E. catax* is a species with particular requirements with regards to its habitat [14,16,19,20]. For example, in Germany and Switzerland, according to the preferred habitats, it is rather a thermo-hydrophilic species often also found in traditionally coppiced forests [15], while in France and Austria, it occurs more frequently in xero-thermophilic habitats [17,21]. It prefers open areas protected from the wind, which is an essential parameter in choosing the oviposition site [15,17,20], due to the reduced mobility of the females [15]. It can be noted that *E. catax* has more or less the same ecological requirements as its host plants [21]. The mandatory characteristic of the habitat of *E. catax* consists in the occurrence of arboreal vegetation comprising mainly *P. spinosa* and *Crataegus* spp. [20]. However, the presence of the host plant is not sufficient for the certain presence of the moth [21]. In Germany, *P. spinosa* is considered to be the main host plant [73]. In Poland, according to [19,31], the main host plant is *P. spinosa,* and *C. monogyna* is rarely used, while [20,31] reported that the species tends to use the pear tree, *Pyrus communis* L., and the black cherry tree, *Prunus serotina* Ehrh. In Austria and Switzerland, the main host plant is *C. monogyna*, followed by *P. spinosa* [15,17,74]. In Spain, in the study of [75], most larvae were found on *C. monogyna* (63%; n = 35), followed by *P. spinosa* (17%) and *Dorycnium pentaphyllum* L. (11%). In the Czech Republic, they feed mostly on *Crataegus* spp., but also on *P. spinosa* [76]. Additionally, there are several reports of nests being present on various species of plants, including: *D. pentaphyllum* L., *Prunus* spp., *Pyrus* spp., *Quercus* spp., *Q. cerrioides* Willk. & Costa, *Q. petrea* (Matt.) Liebl., *Betula* spp., *B. pendula* Roth, *Salix* spp., *S. caprea* L., *Populus* spp., *P. tremula* L., *Ulmus* spp., *U. campestris* L., *U. minor* Mill., *Berberis vulgaris* L., *Rosa* spp., *Fagus sylvatica* L. [16,17,19,21,38,40,48,55,66,73,74,77,78,79,80,81,82,83,84,85,86,87,88]. During this study, *E. catax* nests were found mainly on *P. spinosa*, followed by *C. monogyna*, and very rarely on *Pyrus* ssp., *Prunus tenella* Batsch, *Rosa canina* L., and *Berberis vulgaris* L. Data analysis for the 2011–2016 period revealed a significant preference for *P. spinosa* bushes. Annual data was mostly in favour of *P. spinosa*, excepting the years 2012 and 2015, when a higher preference for *C. monogyna* was noted. These data indicate that *E. catax* successfully uses both species of host plant, even though it manifests an overall stronger preference for one of them. The factors that determine such a preference are probably of extrinsic nature, such as weather conditions, the phenology of the host plant, habitat structure, etc.

*E. catax* generally prefers lower altitudes, but in Eastern Europe and Asia Minor, it has been reported at up to 1500 m altitude [16]. Some authors [75] consider the species to be submontaneous in Spain, where it has been reported at altitudes ranging between 530 and 1500 m. Our study site is situated at an altitude of 400 m, a relatively low elevation, aligning with the distribution pattern of Western European populations.

The solar incidence is important in choosing the oviposition site [15]. The eggs are laid on the sunny side (southern or western) of the bush [20]. The nest increases the absorption surface for the solar rays [15,89,90]; therefore, the choice of oviposition site firstly depends on the position of the bush [21]. According to some authors, there is a preference for southern exposition [79,91], while others noted southern, south-eastern, or eastern exposition [20]. It is important to mention that, in the present study, the cardinal orientation of both the bush and the slope were considered. Our results indicate the highest frequency of nests (65.14%) on the north-western exposition, followed by the northern one (30.63%). This is very likely explained by the need for humidity in the larvae. Thus, the nests on the sunny side of bushes placed on slopes with northern exposition would equally ensure both the necessary insolation (and implicitly the temperature) and the humidity needed for larval development. This positioning of nests and bushes would give the larvae the possibility to benefit from shade during the warmer days. On the other hand, the available shade also depends on the shape, structure, and height of the bushes. With regards to the shape and structure of the bushes, our results have shown a significant preference for grouped bushes, with just 30.12% of nests being placed on isolated bushes. These results are in accordance also with the significant preference of *E. catax* for *P. spinosa*, which forms agglomerations, in contrast with *C. monogyna*, which was generally found isolated.

The average height of the bushes *E. catax* preferred for oviposition was 176.49 ± 54.58 cm. As most of the bushes (64.13%) were between 151 and 200 cm, our results further confirm the previously noted preference for medium-sized bushes [20].

The oviposition height was simply estimated by some authors, while others measured it using a small number of nests. For instance, [17] performed measurements for 39 nests placed on *Crataegus* sp., but without performing any statistical analysis. The nests were placed between 30 and 250 cm, with a medium height of 90 cm. According to [14], the *E. catax* nests can be found at a minimum height of 100 cm. According to [21], the oviposition interval ranges between 50 and 200 cm. In the study of [15], the average oviposition height was 128 cm for nests placed on *C. monogyna* and of 90 cm on *P. spinosa*, ranging between 30 and 170 cm. According to [31], in a recent study conducted in Poland, the nests are distributed between 27 and 248 cm, with most of them in the upper half of the bush and a median 97 cm. In the present study, the average oviposition height was 80.48 ± 34.3 cm. The lowest value (68.74 ± 26.38 cm) was recorded in 2013, while the highest was obtained for 2014 (105.51 ± 38.81 cm). The values from the first three study years are lower compared to the following ones, possibly due to the smaller number of nests from the former years. The average oviposition height on *C. monogyna* (77.26 ± 28.83 cm) was lower compared to *P. spinosa* (85.81 ± 39.15 cm). The insignificant difference between the two species indicates that *E. catax* prefers to constantly lay its eggs within some limits, regardless of the host plant. The global analysis showed that *E. catax* oviposits more frequently in the 61–80 cm (30.63%) and 41–60 cm (24.37%) intervals. The presence of over 50% of nests within the 41–80 cm interval indicates a lower oviposition height compared to previously published literature data. *E. catax* exhibits a low oviposition height, which increases the species’ vulnerability to various human activities, including bush trimming, agricultural field burning, and uncontrolled grazing by sheep and goats—practices that are widespread in both the study area and across Transylvania. Despite the long history of intentional vegetation burning in *E. catax* habitats, dating back at least 3000 years, and the occurrence of natural or accidental fires over tens of thousands of years, our research found no evidence of an adaptive relationship between the height at which eggs are laid and the lethal height of flames.

The species has a single generation per year [19]. The female lays all the eggs once, on a branch of the host plant [15,17,21,45]. According to some authors [20], the female can lay from a few tens to over one hundred eggs, while others [17] indicate it can lay up to 300. Around Vát (Vas County), in Western Hungary, the average number of eggs per nest was estimated to be 55 [71]. The average value obtained during the present study was 186 ± 22 laid eggs/nest. The minimum counted number was 132, while the maximum was 256. The literature data underestimate the number of laid eggs and implicitly the populations of larvae studied by some authors. For instance, the estimation of [71] for larvae in various development stages is underappreciated and faulty, firstly because the authors counted the larvae from the nest’s platform, which are constantly moving. Furthermore, they disregarded the ones that had left the nest and were present on the branches to feed. For the 217 nests that we analysed, 95% of all counted eggs were fertile, showing signs of hatching. The high fertility of females shown by our study is in contradiction with the results obtained by [48], which indicated a fertility of under 60%. However, the study of [48] was performed on a smaller number of laboratory-manipulated nests. This may indicate that laboratory-bred females have a lower fertility compared to the ones that develop in nature.

We consider that all this specific information regarding the ecological requirements of the *E. catax* species in the pastures of Transylvania is crucial for its conservation in Romania, as it provides authorities with the necessary data to establish appropriate and context-specific management measures. Studies conducted by various researchers [14,15,16,19,20,21,46,48] highlight the differences in the species’ ecological requirements depending on factors such as host plant species, climatic conditions, or geographical location.

Habitat loss for *E. catax* through shrub removal is not a local or isolated issue, but rather a common practice employed at the national level. The use of burning as a land-clearing method is inexpensive, and those who employ it, whether out of ignorance or disregard, fail to consider its impact on biodiversity. The intensification of agriculture in Romania, driven in part by European Union subsidies for grazing, has led to an alarming increase in the number of sheep in the country, which, in turn, has resulted in the overutilization of pastures through the removal of shrubs. In contrast, traditional non-intensive grazing practices are essential for maintaining shrubby biotopes in optimal condition, as they foster a mosaic of small shrubs and allow for occasional, controlled disturbances. Such non-intensive grazing, alongside small-scale controlled fires, plays a crucial role in preserving the health of these biotopes, but it is effective only when disturbances remain limited in scale.

### 4.1. Management Measures for the Conservation of E. catax and Its Habitat in Transylvania

Based on the findings of our study and in accordance with the existing literature, the following recommendations were generated:

*Maintaining Shrub Coverage*: Ensure a minimum of 20% shrub coverage in areas with confirmed *E. catax* presence or potential habitat. This coverage is essential for supporting the species’ ecological needs.

*Prohibiting shrub pruning and interventions:* Restrict any pruning or other interventions on shrubs from September to April, the period during which caterpillar nests are visible, to prevent potential damage to the species. If necessary, careful monitoring is needed before an action.

*Conservation of metapopulations*: To preserve *E. catax* metapopulations, maintain or create ecological corridors by keeping shrubs along the strips separating agricultural fields, roadsides, or railway embankments. A mosaic landscape structure ensures the functionality of these metapopulations.

*Avoidance of harmful practices*: Avoid practices such as large scaled and periodically frequently done burning shrubs to clear grasslands in areas where *E. catax* populations are present or the use of insecticide and pest control treatments in areas near *E. catax* habitats. Specifically, avoid the use of insecticides between April 1 and June 15, during the larval development period. In case of natural protected areas, avoid using insecticides by establishing a puffer zone at least 50 m along.

*Relocation of nests in major projects:* In cases where negative habitat impact cannot be avoided due to major infrastructure projects, relocate caterpillar nests to other areas with suitable potential habitat.

*Controlled grazing practices*: Preserve shrub-dominated pastures by implementing controlled grazing, limiting the number of animals per unit area to prevent overgrazing and shrub removal.

*Subsidy programs for shrub maintenance*: Establish or improve or modify an existing subsidy program to incentivize farmers to maintain *Prunus* and *Crataegus* shrubs on their land, which are crucial for the survival of *E. catax.*

*Protection of isolated population:* It is highly important to protect isolated populations from vanishing to save an overall DNA variability. The best practice seems to be establishing the population in captivity and then recolonizing selected sites.

*Dissemination and environmental education activities* targeting the general public, with a particular focus on local communities, stakeholders, and young people. These initiatives can foster greater tolerance towards the hairy larvae of *E. catax*, a species that is less charismatic. The success of conservation initiatives critically depends on the active involvement of local communities and various stakeholders [92,93,94]. While conservation efforts have a global scope, their goals are realized through carefully documented local actions. Engaging the local community should start with clear, easily implementable actions designed to spark curiosity and foster a desire for further knowledge [95].

### 4.2. The Importance of Investigating the Biology and Ecology of Insect Species

Despite the vital role insects play in the stability of our planet, the study of insects is still insufficient, and it is not growing compared to technical humanities studies. Entomology is slowly disappearing from the academic landscape, along with taxonomists [96,97]. Ref. [98] highlights a quote from [99], stating, “*Entomologists are like endangered mammals such as tigers and polar bears in that they and their habitats are on the verge of extinction, and this is likely to have a profound negative effect on science in general*.” This decline reflects a broader trend in which research on nature is increasingly dominated by experimental sciences [100]. The widely publicized decline of taxonomists underscores the urgent need for reinvestment in taxonomy, particularly in entomology [99,100,101].

Insects, the largest group of animals [102,103], remain understudied, with many species disappearing before they can be described and analysed. This situation not only highlights the critical shortage of personal resources but also necessitates increased funding directed towards insect research [104]. Moreover, the decline in the number of entomologists, combined with the knowledge gap, emphasizes the critical importance of ecological studies focused on lesser-known protected insect species. These studies are essential for understanding the specific needs of species and for developing effective land management strategies [5]. Implementing such strategies is crucial not only for the survival of protected species but also for maintaining the biodiversity necessary for ecological balance.

Furthermore, this situation underscores the connection between studying insect biology and ecology and implementing sustainable agricultural practices. As we transition towards agroecology, it is essential to deepen our understanding of insect ecology to enhance biodiversity and ecosystem services.

### 4.3. Collaborative Efforts for Sustainable Outcomes

Ref. [105] argued that, although agricultural landscapes have the potential to offer extensive habitats for insects, the prevailing intensive farming practices are not insect-friendly, necessitating the implementation of more effective solutions. We are fully aware that developing these solutions requires concrete data derived from detailed species ecology studies.

At the European level, there is a strong push for a transition towards agroecology. Agroecology aims not only to boost agricultural productivity but also to enhance biodiversity and ecosystem services [106,107]. To align these practices with the objectives established by the [108], it is crucial to gain a deeper understanding of the biology and ecology of insects. Such knowledge would enable the development of specific actions, including educational programs addressed to diverse categories of learners, to ensure that insects continue to fulfil essential functions like pollination and pest control, thereby reducing reliance on pesticides and supporting other ecosystem services [98]. Furthermore, agroecological practices can aid in restoring natural habitats, which are vital for the survival of numerous insect species, including the protected species *E. catax.*

## 5. Conclusions

*E. catax*, protected at the European level, serves as an umbrella species, meaning that its conservation can indirectly protect a wide array of other species within the same habitat. The data obtained from ecological studies of *E. catax* offer a crucial starting point for understanding the species’ ecology and habitat requirements. Protecting this species inherently means preserving its habitat in an appropriate shape, which includes a mosaic of small *Prunus* and *Crataegus* shrubs at least at north and northwest slopes. These shrubs not only support *E. catax* but also provide shelter for numerous other species, such as birds and small mammals. Thus, by safeguarding the habitat of this moth, we simultaneously protect a diverse range of other animal/plant species.

We consider that the integration of public education and research-informed, well-designed policies is crucial for the success of conservation efforts. Educating the public about the importance of various insect species and their roles in ecosystems can foster a culture of conservation. By highlighting the interconnectedness of species and the broader ecological benefits of protecting *E. catax*, we can encourage public support for conservation initiatives. Implementing policies that incentivize sustainable practices ensures that these efforts are practical and beneficial for all stakeholders involved. Efforts in the direction of designing interdisciplinary curricular content for ecological educational programs addressing insects and their roles in ecosystems are currently being made in Romania [109].

Moreover, the data obtained from ecological studies are invaluable not only for creating suitable land management strategies but also for public education. Bridging the gap between scientific research and public awareness fosters a deeper understanding and appreciation of biodiversity, ultimately driving more effective conservation efforts. For instance, citizen science and ecological education programs can engage local communities in monitoring and protecting *E. catax* and its habitat. This shared responsibility transforms conservation from a specialized endeavour into a collective effort, changing the prevailing aesthetic perception of biotopes and enhancing the prospects of long-term ecological sustainability.

By combining education, policy, and community engagement, we can create a robust framework for preserving biodiversity and securing the health of our ecosystems for future generations. The conservation of *E. catax,* therefore, serves as a model for how integrated approaches can effectively protect biodiversity—here built on the protection of the still common bush habitat, which is generally considered useless, unsightly, or even undesirable by common people. Protecting this species of moth not only supports its survival, but also ensures the preservation of the complex web of life within its habitat, highlighting the broader ecological significance of targeted conservation efforts.

## Figures and Tables

**Table 1:** The frequency and 95% CI of nests according to host plant species. To determine the preference for the host plant was used one-sample chi square test, considering an expected ratio of 58% in favour of *P. spinosa* according to host availability.

	*Prunus spinosa* L.		*Crataegus monogyna* Jacq.		χ^2^ (df = 1)	*p*
	Frequency (%)	95% CI	Frequency (%)	95% CI		
2011	70.75	64.13–76.78	29.25	23.22–35.87	27.19	<0.0001
2012	28.13	22.34–34.50	71.88	65.50–77.66	162.12	<0.0001
2013	73.86	63.41–82.66	26.14	17.34–35.59	16.99	<0.0001
2014	63.64	45.12–79.60	36.36	20.40–54.88	0.55	0.45
2015	33.33	9.92–65.11	66.67	34.89–90.08	4.28	0.03
2016	77.27	54.62–92.18	22.73	7.82–45.37	4.6	0.03
Total	54.15	50.11–58.12	45.85	41.88–49.89	7.03	0.007

**Table 2:** The frequency and 95% CI of the nests of *E. catax* in relation to the shape and structure of the host plant. To determine the preference for the shape and structure of host plant was used one-sample chi square test, considering an expected ratio of 50:50 for expected values.

	Isolated Bush		Grouped Bushes		χ^2^ (df =1)	*p*
	Frequency (%)	95% CI	Frequency (%)	95% CI		
2011	26.42	20.61–32.89	73.58	67.11–79.39	94.34	<0.0001
2012	30.80	24.83–37.30	69.20	62.70–75.17	66.03	<0.0001
2013	45.45	34.80–56.42	54.55	43.58–65.20	1.84	0.2
2014	27.27	13.30–45.52	72.73	54.48–86.70	13.64	0.0002
2015	25	5.49–57.11	75	42.81–94.51	6	0.0143
2016	4.55	0.12–22.84	95.45	77.16–99.88	33.36	<0.0001
Total	30.12	26.56–33.24	69.88	66.06–73.44	188.48	<0.0001

**Table 3:** The frequency and 95% CI of cardinal orientation points. To determine the preference for the cardinal orientation was used one-sample chi square test, considering an equal proportion for expected values.

	N		NW		SW		W		χ^2^ (df = 3)	*p*
	%	95% CI	%	95% CI	%	95% CI	%	95% CI		
2011	56.66	48.70–62.46	36.79	30.29–43.67	3.77	1.64–7.30	3.77	1.64–7.30	223.89	<0.0001
2012	12.50	8.47–17.56	87.50	82.44–91.53	0	-	0	-	634.66	<0.0001
2013	13.64	7.25–22.61	85.23	76.06–91.89	1.14	0.03–6.17	0	-	232.36	<0.0001
2014	30.30	15.59–48.71	69.70	51.59–84.41	0	-	0	-	57.65	<0.0001
2015	33.33	9.92–65.11	50	21.09–78.91	16.67	2.09–48.41	0	-	8.88	0.03
2016	40.91	20.71–63.65	31.87	13.86–54.87	27.27	10.73–50.22	0	-	10.9	0.01
Total	30.63	27.04–34.46	65.14	61.22–68.88	2.88	1.80–4.56	1.35	0.69–2.65	884.45	<0.0001

## Data Availability

The original contributions presented in the study are included in the article, further inquiries can be directed to the corresponding authors.

## Supplementary Materials

The following supporting information can be downloaded at: https://www.mdpi.com/article/10.3390/insects15100794/s1, Table S1: The frequency and 95% CI of height intervals of host plants; Table S2: The average height of the host plant; Table S3: The overall frequency and 95% CI of oviposition intervals; Table S4: The frequency and 95% CI of oviposition intervals on *Prunus spinosa*; Table S5: The frequency and 95% CI of oviposition intervals on *Crataegus monogyna;* Table S6: The average oviposition height.
