# Supplementary material for: Multi-Annual Study of Eriogaster catax (Linnaeus, 1758) (Lepidoptera, Lasiocampidae) Oviposition Strategy in Transylvania’s Largest Population: Key Insights for Species Conservation and Local Land Management
Source: Insects. 2024 Oct 12;15(10):794. doi: 10.3390/insects15100794 (PMC11508962; doi:10.3390/insects15100794)
Supplement: Supplementary file 1 [file insects-15-00794-s001.zip › insects-3218089-supplementary.pdf]

Table S1. The frequency and 95% CI of height intervals of host plants. Maximum values are highlighted in grey.

		2011	2012	2013	2014	2015	2016	Total
51-100 cm	%	9.91	7.59	5.68	12.12	0	4.12	8.14
	95% CI	8.38-11.67	4.48-11.87	4.11-7.80	3.40-28.20	-	0.12-22.84	6.19-10.62
101-150 cm	%	14.62	13.84	14.77	12.12	16.67	4.12	13.87
	95% CI	12.79-16.67	9.60-19.07	12.19-17.79	3.40-28.20	2.09-48.41	0.12-22.84	11.32-16.90
151-200 cm	%	67.45	65.63	67.05	42.42	33.33	54.55	64.13
	95% CI	64.83-69.97	59.01-71.82	63.24-70.74	25.48-60.78	9.92-65.11	32.21-75.61	60.18-67.89
201-250 cm	%	3.3	12.05	5.68	18.18	50	13.64	9.14
	95% CI	2.45-4.43	8.10-17.05	4.11-7.80	6.98-35.46	21.09-78.91	2.91-34.91	7.07-11.73
251-300 cm	%	0	0.89	6.82	3.03	0	9.09	1.86
	95% CI	-	0.11-3.19	5.08-9.09	0.08-15.76	-	1.12-29.16	1.04-3.30
301-350	%	4.72	0	0	9.09	0	13.64	2.71
	95% CI	3.68-6.02	-	-	1.92-24.33	-	2.91-34.91	1.67-4.35
351-400 cm	%	0	0	0	3.03	0	0	0.17
	95% CI	-	-	-	0.08-15.76	-	-	0.03-0.95

Table S2. The average height of the host plant

An	*Prunus spinosa* L.		*Crataegus monogyna* Jacq.		Total	
	**Average (cm)**	**Stdev**	**Average (cm)**	**Average (cm)**	**Stdev**	**Average (cm)**
2011	169.55	55.79	148.44	29.52	163.77	50.81
2012	172.46	41.29	161.24	34.23	164.4	36.61
2013	179.07	35.82	168.69	44.14	176.36	38.18
2014	192.86	89.99	198.33	58.16	194.85	78.94
2015	212.5	41.13	200	41.32	204.17	39.82
2016	215.88	72.98	236	58.99	220	69.2
Total	176.49	54.58	163.33	39.22	170.55	48.66

Table S3. The overall frequency and 95% CI of oviposition intervals. Maximum values are highlighted in grey.

		**2011**	**2012**	**2013**	**2014**	**2015**	**2016**	**Total**
**0-20 cm**	%	0.47	0	0	0	0	0	0.17
	95% CI	0.01-2.60	-	-	-	-	-	0.03-0.95
**21-40 cm**	%	9.91	5.36	4.55	0	0	0	6.26
	95% CI	6.24-14.74	2.80-9.17	1.25-11.23	-	-	-	4.58-8.51

41-60 cm	%	16.51	29.91	37.5	12.12	16.67	13.64	24.37
	95% CI	11.78-22.21	23.99-36.37	27.40-48.47	3.40-28.20	2.09-48.41	2.91-34.91	21.08-27.98
61-80 cm	%	25.94	34.38	35.23	27.27	33.33	40.91	30.63
	95% CI	25.18-32.39	28.18-40.99	25.34-46.14	13.30-45.52	9.92-65.11	20.71-63.65	27.04-34.46
81-100 cm	%	24.06	14.73	14.77	18.18	18.18	27.27	19.12
	95% CI	18.47-30.39	10.36-20.06	8.11-23.94	6.98-35.46	2.28-51.78	10.73-50.22	16.15-22.49
101-120 cm	%	11.79	8.93	5.68	15.15	8.33	18.18	10.15
	95% CI	7.78-16.91	5.54-13.45	1.87-12.76	5.11-31.9	0.21-38.48	5.19-40.28	7.97-12.85
121-140 cm	%	3.77	5.36	1.14	6.06	16.67	0	4.23
	95% CI	1.64-7.3	2.8-9.17	0.03-6.16	0.74-20.23	2.09-48.41	-	2.88-6.17
141-160 cm	%	0.94	0.45	0	3.03	8.33	0	0.85
	95% CI	0.11-3.37	0.01-2.46	-	0.08-15.76	0.21-38.48	-	0.36-1.97
161-180 cm	%	3.3	0.89	0	18.18	0	0	2.88
	95% CI	1.34-6.68	0.11-3.19	-	6.98-35.46	-	-	1.80-4.56
181-200 cm	%	1.42	0	0	0	0	0	0.51
	95% CI	0.29-4.08	-	-	-	-	-	0.17-1.48
201-220 cm	%	0	0	0	0	0	0	0
	95% CI	-	-	-	-	-	-	-
221-240 cm	%	0.94	0	1.14	0	0	0	0.51
	95% CI		-	0.03-6.16	-	-	-	0.17-1.48
241-260 cm	%	0.47	0	0	0	0	0	0.17
	95% CI	0.01-2.60	-	-	-	-	-	0.03-0.95
261-280 cm	%	0.47	0	0	0	0	0	0.17
	95% CI	0.01-2.60	-	-	-	-	-	0.03-0.95

Table S4. The frequency and 95% CI of oviposition intervals on *Prunus spinosa*. Maximum values are highlighted in grey.

		2011	2012	2013	2014	2015	2016	Total
21-40 cm	%	10.39	3.17	6.15	0	0	0	7.1
	95% CI	6.06-16.32	0.39-11	1.7-15.01	-	-	-	4.78-10.43
41-60 cm	%	13.64	28.57	36.92	4.76	0	17,65	19.75
	95% CI	8.64-20.09	17.89-41.35	25.28-49.8	0.12-23.82	-	3.80-43.43	15.78-24.43
61-80 cm	%	24.68	46.03	30.77	19.05	50	47.06	30.86
	95% CI	18.09-32.06	33.39-53.06	19.91-43.45	5.45-41.91	6.76-93.42	22.98-72.19	26.08-36.09
81-100 cm	%	25.32	12.7	18.46	19.05	0	23.53	20.06
	95% CI	18.67-32.95	5.65-23.5	9.92-30.03	5.45-41.91	-	6.81-49.90	16.06-24.76
101-120 cm	%	14.29	6.35	6.15	14.29	25	11.76	11.11
	95% CI	9.17-20.83	1.76-15.47	1.7-15.01	3.05-36.34	0.63-80.59	1.46-36.44	8.13-15.00
121-140 cm	%	1.95	0	1.54	4.76	25	0	1.54
	95% CI	0.40-5.59	-	0.04-8.28	0.12-23.82	0.63-80.59	-	0.66-3.56

**141-160 cm**	%	1.3	0	0	4.76	0	0	0.93
	95% CI	0.16-4.61	-	-	0.12-23.82	-	-	0.32-2.69
**161-180 cm**	%	3.9	3.17	0	33.33	0	0	4.63
	95% CI	1.44-8.29	0.39-11	-	14.59-56.97	-	-	2.83-7.50
**181-200 cm**	%	1.95	0	0	0	0	0	0.93
	95% CI	0.40-5.59	-	-	-	-	-	0.32-2.69
**201-220 cm**	%	0	0	0	0	0	0	0
	95% CI	-	-	-	-	-	-	-
**221-240 cm**	%	1.3	0	0	0	0	0	0.62
	95% CI	0.16-4.61	-	-	-	-	-	0.17-2.22
**241-260 cm**	%	0.65	0	0	0	0	0	0.31
	95% CI	0.02-3.56	-	-	-	-	-	0.05-1.75
**261-280 cm**	%	0.65	0	0	0	0	0	0.31
	95% CI	0.02-3.56	-	-	-	-	-	0.05-1.75

Table S5. The frequency and 95% CI of oviposition intervals on *Crataegus monogyna.* Maximum values are highlighted in grey.

		**2011**	**2012**	**2013**	**2014**	**2015**	**2016**	**Total**
**0-20 cm**	%	1.72	0	0	0	0	0	0,38
	95% CI	0,04-9,24	-	-	-	-	-	0,01-2,11
**21-40 cm**	%	6,9	6,25	0	0	0	0	5,34
	95% CI	1,91-16,73	3,04-11,19	-	-	-	-	2,95-8,80
**41-60 cm**	%	24,14	30,63	42,11	25	25	0	29,01
	95% CI	13,87-37,17	23,59-38,39	20,25-66,50	5,49-57,19	3,19-65,09	-	23,59-34,91
**61-80 cm**	%	31,03	30	42,11	8,33	25	20	29,01
	95% CI	19,54-44,54	23,02-37,74	20,25-66,50	0,21-38,48	3,19-65,09	0,51-71,64	23,59-34,91
**81-100 cm**	%	20,69	15	10,53	33,33	25	40	17,94
	95% CI	11,17-33,35	9,85-21,49	1,30-33,14	9,92-65,11	3,19-65,09	5,27-85,34	13,49-23,13
**101-120 cm**	%	5,17	10	0	16,67	0	40	8,78
	95% CI	1,08-14,38	5,82-15,73	-	2,09-48,41	-	5,27-85,34	5,65-12,88
**121-140 cm**	%	8,62	7,5	0	8,33	12,5	0	7,22
	95% CI	2,86-18,68	3,94-12,73	-	0,21-38,48	0,32-52,65	-	4,41-11,05
**141-160 cm**	%	0	0,63	0	0	12,5	0	0,76
	95% CI	-	0,02-3,43	-	-	0,32-52,65	-	0,09-2,73
**161-180 cm**	%	1,72	0	0	8,33	0	0	0,76
	95% CI	0,04-9,24	-	-	0,21-38,48	-	-	0,09-2,73
**181-200 cm**	%	0	0	0	0	0	0	0
	95% CI	-	-	-	-	-	-	-
	%	0	0	0	0	0	0	0

**201-220 cm**	95% CI	-	-	-	-	-	-	-
**221-240 cm**	%	0	0	5,26	0	0	0	0,38
	95% CI	-	-	0,13-26,03	-	-	-	0,01-2,11

Table S6. The average oviposition height

	Prunus spinosa **L.**		***Crataegus monogyna*** **Jacq.**		Total	
	**Average (cm)**	**Stdev**	**Average (cm)**	**Average (cm)**	**Stdev**	**Average (cm)**
2011	88.24	44.48	75.467	29.276	84.504	41.12
2012	74.936	24.255	76.161	26.387	75.816	25.757
2013	67.292	22.246	72.826	35.906	68.738	26.382
2014	111.238	40.615	95.5	34.809	105.515	38.815
2015	98.5	24.117	86.875	34.909	90.75	31.095
2016	79.117	18.475	162	54.037	98	45.6
Total	85.81	39.154	78.372	30.768	82.298	35.605
